# Fatal Multiorgan Failure Syndrome in a *Strongyloides*-HTLV-1 Coinfected Patient, after Treatment with Ivermectin

**DOI:** 10.1155/2021/5554810

**Published:** 2021-09-10

**Authors:** Emmanuelle Guérin, Paule Poirier, Marine Nervo, Christophe Le Terrier

**Affiliations:** ^1^Intensive Care Unit, University Hospital of Martinique, Fort-de-France, France; ^2^Department of Pathology, Saint-Louis University Hospital, AP-HP, Paris University, Paris, France; ^3^Intensive Care Unit, University Hospitals of Geneva, Geneva, Switzerland

## Abstract

Because of its characteristic features of autoinfection, the parasitic nematode *Strongyloides stercoralis* can infect patients for years. An acceleration of its autoinfective cycle can be triggered by human T-lymphotropic virus-1 (HTLV-1) infection, mainly by the deviation of the protective Th2- to Th1-type immune response and can lead to severe disease by dissemination of *Strongyloides stercoralis* larvae carrying intestinal bacteria to multiple organs. Meningitis caused by enteric Gram-negative bacteria is a potentially fatal complication of disseminated strongyloidiasis. Herein, we present the case of a *Strongyloides*-HTLV-1 coinfected patient, admitted for *E. coli* meningitis. One day after initiation of ivermectin, the patient developed significant *S. stercoralis* dissemination, complicated by multiorgan failure syndrome, and died from neurological failure. While the initial clinical scenario of our case has already been well described in the literature, its course after antihelminthic treatment initiation remains unclear and needs to be discussed.

## 1. Introduction

*Strongyloides stercoralis*, a nematode endemic in tropical and subtropical regions, is responsible for chronic, mostly asymptomatic, infections, because of its ability to autoinfect its host. Indeed, after the host's skin penetration, the filariform larva migrates through the bloodstream to the lungs, passes through the alveoli, ascends the bronchial and tracheal trees, and is eventually swallowed. After maturation of its eggs in the small intestine, these can be excreted in the stool or penetrate the intestinal mucosa to join the bloodstream perpetuating the cycle.

Sharing some endemic regions with *Strongyloides stercoralis*, HTLV-1 is an oncoretrovirus which can cause an aggressive malignancy named adult T-cell leukemia/lymphoma (ATLL) and a progressive chronic inflammatory neurological disease called HTLV-1-associated myelopathy/tropical spastic paraparesis (HAM/TSP). In patients infected with HTLV-1 and ATLL, opportunistic infections can be observed due to immunosuppression.

Moreover, studies support that *Strongyloides*-HTLV-1 coinfection may change the course of either infection. Host immune Th2 response is a typical effective defense mechanism to helminthiases. In case of coinfection with HTLV-1, the proliferation of viral-infected cytokine type 1 secreting CD4+ T lymphocytes shifts the Th1/Th2 balance to the Th1 response. Indeed, the Th1-induced high production of interferon gamma (IFN-*γ*) can downregulate Th2 cells by decreasing the secretion of cytokines IL-4, IL-5, and IL-13, resulting in reduction in total and specific IgE antibodies against *S. stercoralis* and failure of proliferation and activation of eosinophils [1]. The host cannot produce an effective immune antiparasitic response, allowing a large increase in worm burden and a risk of developing disseminated infection, with the migration of the larvae outside the gastrointestinal-pulmonary autoinfective cycle [2].

On the other side, *S. stercoralis* infection might accelerate the onset of HTLV-1-associated diseases such as ATLL by stimulating the oligoclonal proliferation of HTLV-1-infected cells inducing potential additional cellular mutations enhancing the likelihood of malignant change [3].

Our case illustrates the risk of occurrence of secondary bacterial infections as Gram-negative meningitis in case of disseminated infection, in a *Strongyloides*-HTLV-1 coinfected patient. Furthermore, the patient developed multiorgan failure syndrome after ivermectin initiation, and its neurological outcome was unfavorable. The mechanisms for these evolutions are not explained and must be discussed.

## 2. Case

A 61-year-old Caribbean man was admitted to the intensive care unit (ICU) for impaired consciousness with a Glasgow score of 13, stiff neck, and fever of 102.2°F. He did not present any medical condition, except for a 3-month abdominal history with severe diarrheas and 33-pound weight loss explored by his general practitioner (GP). His white blood cell count was 13.10/*μ*L with 10.42/*μ*L neutrophils, 1.78/*μ*L lymphocytes, and 0.01/*μ*L eosinophils.

**Figure 1:**
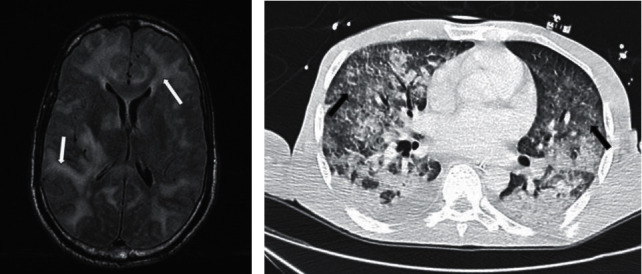
Imaging findings. (a) Cerebral MRI (axial, T2/FLAIR) showing cerebral edema compressing ventricles and gadolinium-enhancing T2/FLAIR white matter and basal ganglia hyperintensity, evoking acute necrotizing encephalopathy. (b) Chest CT scan showing bilateral ground-glass opacities.

Empiric anti-infective therapy for community-acquired meningitis, including cefotaxime 300 mg/kg/d, amoxicillin 200 mg/kg/d, acyclovir 10 mg/kg/d, and dexamethasone 10 mg/6 h, was initiated. Cerebral magnetic resonance imaging (MRI) showed severe cerebral edema compressing ventricles and gadolinium-enhancing T2/FLAIR white matter and basal ganglia hyperintensity, revealing acute necrotizing encephalopathy (ANE) (Figure 1(a)). The day after ICU admission, immune status checking revealed HTLV-1 seropositivity. CD4+ T lymphocyte count was not evaluated. A lumbar puncture demonstrated pleiocytosis with more than 4500 cells per mm^3^ (87% polynuclear neutrophils), lactate 15.80 mmol/L, protein 3.67 g/L, and glucose in the normal range, in the cerebrospinal fluid (CSF). Blood and CSF cultures were positive for *E. coli*.

**Figure 2:**
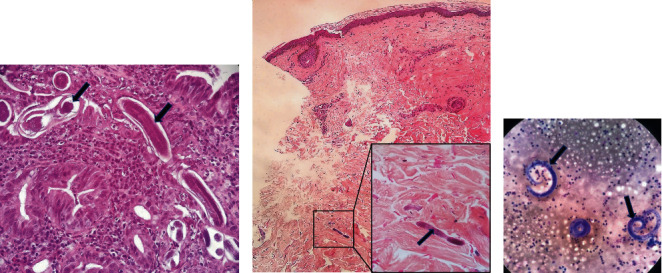
Histological examination showing *Strongyloides stercoralis* larvae. (a) Duodenal biopsy: duodenal crypts distended by numerous larvae and adult worms. (b) Purpuric lesion transcutaneous biopsy: deep dermis location of filariform larvae between collagen bundles. (c) Bronchoalveolar aspiration: inflammation and filariform larvae.

**Figure 3:**
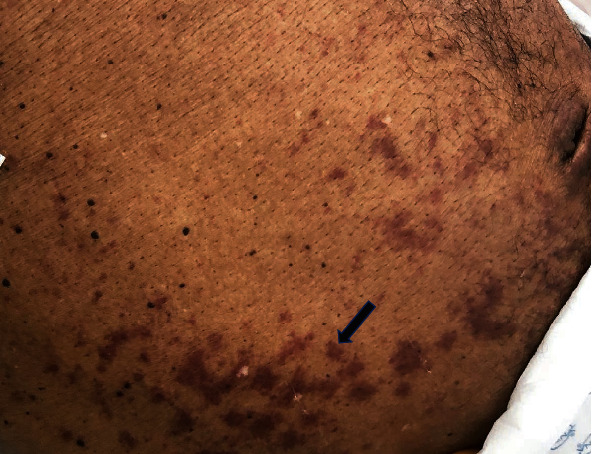
Abdominal physical examination showing periumbilical purpuric lesions.

Enhanced abdominal computed tomography (CT) was performed to detect an infectious focus for Gram-negative bacteremia and showed nonspecific wall thickening in the jejunum. The access to GP's investigations revealed untreated *S. stercoralis* infection diagnosed by duodenal biopsy 2 months before (Figure 2(a)). A periumbilical, extending to the flanks, purpuric rash appeared after admission (Figure 3). Punch biopsy of this rash confirmed parasitic chronic infection, showing filariform larvae between collagen bundles in the dermis (Figure 2(b)).

After one day of treatment for community-acquired meningitis, notably including a total of 40 mg of dexamethasone, the patient was treated with ivermectin 200 mcg/kg orally daily during two days for disseminated strongyloidiasis and cefotaxime 200 mg/kg intravenous for *E. coli* meningitis.

One day after ivermectin initiation, the patient presented coma and acute respiratory failure requiring endotracheal intubation followed by severe hemodynamic instability. The patient developed abundant hemoptysis with alveolar hemorrhage evolving toward acute respiratory distress syndrome. Chest CT scan showed bilateral ground-glass opacities (Figure 1(b)). Numbers of larvae of *S. stercoralis* in bronchoalveolar lavage were detected (Figure 2(c)). Eosinophilia appeared in the days that followed and stayed elevated, until 3.76/*μ*L eosinophils.

Despite hemodynamic and respiratory improvements, the patient suffered severe neurological impairment (Glasgow score of 7). Repeat MRI showed a global stability of the lesions. Several electroencephalograms (EEG) showed unreactive slow wave activity.

Control lumbar puncture realized 4 days after antibiotic initiation showed CSF sterilization and 2300 cells per mm^3^ (24% neutrophils and 75% lymphocytes). Stool and sputum smears showed persistence of *Strongyloides* larvae.

After three weeks of intensive care, no neurological improvement was noticed, and care was withdrawn. The patient died and no autopsy was performed.

## 3. Discussion

Chronic strongyloidiasis is underestimated because it is asymptomatic or mildly symptomatic with unspecific signs such as abdominal pain and diarrhea at clinical presentation. Eosinophilia might be the only laboratory finding. *Larva currens*, a linear fleeting and moving urticaria, pathognomonic skin eruption of chronic strongyloidiasis [4], and eosinophilia, especially in the case of coinfection with HTLV-1 which impairs the proliferation and the activation of eosinophils, can be missing as illustrated in our case. Health-care providers, particularly in endemic regions, should be familiar with this infection and prompt to treat it in order to prevent severe presentations such as SHS and disseminated strongyloidiasis with the parasite's intestinal wall invasion, facilitating enteric bacterial translocation, leading to bacteremia and potentially septic shock [5]. In our case, the diagnosis of *S. stercoralis* infection was realized by duodenal biopsy two months before, and the patient was unfortunately lost to follow-up without receiving antihelminthic treatment. The untreated *Strongyloides*-infected patient presented Gram-negative meningitis, which has also been classically described in the case of disseminated strongyloidiasis [6, 7]. This underlying diagnosis must be systematically evoked and explored in the case of Gram-negative meningitis, particularly in HTLV-1 careers.

Corticosteroids and HTLV-1 are the most common risk factors of SHS [8]. It means SHS can be prevented by deworming before starting corticosteroid therapy and by reciprocal screening of *S. stercoralis* and HTLV-1 infection once one of these two infections is detected, especially in areas of high prevalence of both infections, such as Japan, South America, Africa, and Australia [9]. By shifting the immune responses from Th2 to Th1, HTLV-1 infection alters the immunological defense against strongyloidiasis. In our case, immunological data have unfortunately not been checked due to rapid worsening of the patient's condition. It would have been interesting and should be recommended in the case of *Stongyloide*s-HTLV-1 coinfection to screen cytokine levels (IFN-*γ*, IL-4, IL-5, and IL-13), serum parasitic specific and total IgE levels, CD4+ T lymphocyte count, and proviral load.

Usually, chronic strongyloidiasis is treated by a regimen of two single doses of 200 *μ*g/kg ivermectin, given 2 weeks apart. Single-dose ivermectin has recently been evaluated successfully for the treatment of nondisseminated strongyloidiasis in a randomised controlled superiority trial [9]. Nevertheless, the medical community should be aware that deworming in hyperinfected patients is both essential and at risk of worsening the patient's condition as has been already reported [10].

A possible explanation of this treatment failure is the possibility of oral ivermectin inefficiency, as suggested by persistence of larvae in the stool in our case. Indeed, impairment of antihelminthic agents' action has been described in the case of *Strongyloides*-HTLV-1 coinfection [11]. The patient did not present any signs of ileus or bowel obstruction which could have suggested ivermectin malabsorption. In this situation, the safety and efficacy of subcutaneous ivermectin have been reported [12].

Another explanation is a significant dissemination of *S. stercoralis* which paradoxically occurs after antihelminthic treatment initiation. The sudden respiratory impairment of the patient with the detection of numbers of *S. stercoralis* larvae in bronchoalveolar lavage and the appearance of eosinophilia after ivermectin initiation are consistent with this hypothesis, even if no bronchoscopy with bronchoalveolar lavage was performed before treatment administration due to the absence of respiratory impairment.

The last explanation for this abrupt dissemination of *S. stercoralis* could be a synergism of HTLV-1 infection and the low-dose dexamethasone administrated for treatment of the meningitis, without preventive deworming, as corticosteroids are known as accelerators of the autoinfective cycle, even with a low dose of corticosteroids [13]. Combination of corticosteroids and HTLV-1 triggering hyperinfection has already been described in case reports [14].

Even if we cannot conclude that the dissemination of *S. stercoralis* is due to ivermectin or corticosteroids, it seems clear that the general condition of the patient, altered by 3 months of chronic strongyloidiasis leading to malnutrition, contributed to this complication.

Despite *E. coli* meningitis management, the patient did not recover neurologically. The first MRI revealed multifocal, symmetric brain lesions involving both the gray matter and the white matter, with a remarkable topographic distribution including thalami and the cerebellum, consistent with ANE, which usually develops secondary to viral infections [15]. Typical *E. coli* brain damage is mostly ventriculomegaly [16]. The patient's neurological impairment seems to be secondary to multiorgan failure syndrome, despite the lack of ischemic lesions on the second MRI. Another explanation could be posttreatment cerebral *S. stercoralis* dissemination, even if it has not been described in the literature and no parasites have been found in the control lumbar puncture.

In severe manifestations of *Strongyloides stercoralis* infection, including hyperinfection syndrome and disseminated strongyloidiasis, the mortality rate is up to 85%. Even the presence of peripheral eosinophilia during hyperinfection seems to be associated with a better outcome [6, 17, 18]; factors such as delayed diagnosis, concomitant immunosuppression, and bacteremia are linked to higher mortality [19]. In our case, late diagnosis of strongyloidiasis, HTLV-1 coinfection responsible for immunosuppression and *E. coli* meningitis with bacteremia, appeared to predict a bad outcome for the patient.

## 4. Conclusion

Regular appropriate prevention and prompt treatment of *S. stercoralis* infections, especially in at-risk of disseminated strongyloidiasis patients such as HTLV-1 coinfected careers or in the case of corticosteroid therapy, are essential to avoid serious clinical presentations and their consequences such as Gram-negative meningitis. Early and accurate diagnosis is challenged by the fact that *Strongyloides* and HTLV-1 infections might stay asymptomatic for years before potentially developing their own dramatic presentation.

Clinical worsening after ivermectin initiation may occur in the case of SHS, with significant *S. stercoralis* dissemination, complicated with multiorgan failure syndrome. Mechanisms of this treatment's paradoxical effect remain unclear and must be explored. In the meantime, another route of administration or fractionated doses of the ivermectin regimen should be studied in the SHS.

## Data Availability

All data used to support the findings of this study are available from the corresponding author upon request.
